# Dignified Resources and Coping Strategies During the COVID-19 Pandemic: a Qualitative Study of Racially and Economically Marginalized Communities

**DOI:** 10.1007/s40615-023-01824-x

**Published:** 2023-10-16

**Authors:** Alice Guan, Tessa Cruz, Jamaica Sowell, Brenda Mathias, Analena Hope Hassberg, Salma Shariff-Marco, Antwi Akom, Mindy C. DeRouen

**Affiliations:** 1 Department of Epidemiology and Biostatistics | University of California, San Francisco, USA; 2 The Social Innovation and Urban Opportunity Lab, Streetwyze | UCSF & San Francisco State University, San Francisco, Oakland, CA USA; 3 Roots Community Health Center, Oakland, CA USA; 4 Department of Sociology | California State University, Los Angeles, CA USA; 5 Department of Africana Studies | San Francisco State University, San Francisco, USA

**Keywords:** COVID-19, Community-based participatory research, Qualitative research, Health disparities

## Abstract

**Introduction:**

Amid the spread of the novel coronavirus (COVID-19), racially and economically marginalized communities experienced a disproportionate burden of disease and social consequences (e.g., unemployment, increased exposure). This study seeks to understand strategies that these communities employed to cope with unequal burdens of the pandemic.

**Methods:**

We utilized qualitative data collected between 2020 and 2021 from a mobile mapping platform designed to facilitate real-time, geocoded data collection on individual’s experiences and perceptions of their neighborhoods. Reports were iteratively coded by an academic researcher and community partner. We employed an inductive approach to analysis, which allowed findings to emerge organically without constraint of researcher hypotheses.

**Results:**

A total of 19 respondents (14 under the age of 45, 16 non-White, 15 with less than half a year of emergency savings) provided 236 qualitative reports. Participants described innovative strategies for exchanging resources as a means of informally networking and building community, the importance of tailored programming (e.g., for specific racial/ethnic groups) in fostering belonging and comfort, and the importance of two specific dimensions of services—interactions with service providers and the quality of goods or services—in providing dignified care.

**Discussion:**

Amidst exacerbated racial and economic disparities emerging from the COVID-19 pandemic, our study highlights the need for investment in mutual aid, the importance of tailored services and support, and promoting dignity in social services. As other macro-level social stressors become more prevalent as the pandemic continues, these findings can inform how we examine and address them.

**Supplementary Information:**

The online version contains supplementary material available at 10.1007/s40615-023-01824-x.

## Introduction

While COVID-19 has had immediate and dire consequences on the health and well-being of the global collective, the pandemic has inequitably burdened racially marginalized and socioeconomically vulnerable populations. The distribution of COVID-19 cases and deaths has been higher among Black and Hispanic Americans compared to White individuals [1–3]. A systematic review of 72 studies found that Black, Hispanic, and other non-White individuals had higher prevalence, hospitalization, and mortality due to COVID-19 compared with White individuals [4], which remained after accounting for publication bias. Additionally, racially segregated, and economically marginalized counties have been found to have higher COVID-19 mortality rates [5]. Scholars have implicated racial capitalism as a fundamental cause of health disparities and inequities during the pandemic [6], which Link and Phelan theorized to be root social causes that influence health through multiple pathways, impact access to resources, and are reproduced over time through systems and structures [7]. One such pathway through which racial capitalism has exacerbated COVID-19 disparities is through previously existing inequalities resulting from structural vulnerability at the neighborhood level (e.g., through historical racial and socioeconomic segregation) [8, 9]. Poor communities are more likely to be overcrowded, thus increasing the likelihood of community transmission among residents [10]. Moreover, the overrepresentation of racially and economically marginalized communities in low-wage and front line workforces increased residents’ exposure to the virus [11]. Altogether, the disproportionate burden of disease and unique lived experiences within these communities warrant an examination of resources and solutions that reduced the negative impact of the pandemic for residents. Such studies are critical for developing scalable interventions to reduce the unequal burden that vulnerable communities face in the presence of macro-level stressors (e.g., climate stress, recession) more broadly.

Qualitative insights can provide a more comprehensive understanding of individuals’ lived experiences during the pandemic. Existing qualitative research on impacts of the pandemic has focused on experiences of patients, healthcare professionals [12–14], college students [15, 16], and older adults [17–20]. One study focusing specifically on the experiences of Black people in the USA and the UK found that while few described hindrances to accessing vaccination, mistrust in the healthcare system continued to be a barrier, highlighting the need to strengthen underlying social inequalities [21]. These themes were further described in another study with Black Americans with low vaccination intentions, in which participants detailed structural barriers to access and medical mistrust due to historical and systematic racism as reasons for their low confidence [22]. Other qualitative studies described that ethnic minorities and vulnerable groups were uncomfortable participating in COVID-19 vaccine trials due to mistrust and the need for cultural support and transparency [23]. Given the prevailing descriptions of structural barriers faced by racially marginalized communities, our study aims to extend the literature by providing qualitative descriptions of resources and strategies utilized by residents of such communities in response to pandemic burdens. These descriptions offer valuable insights into how vulnerable communities have maintained health and well-being, potentially pointing to sustainable strategies to address ongoing and emergent needs.

## Methods

### Sample and Data Collection

This manuscript utilizes qualitative data from a mixed-methods study that sought to identify community resources and needs during the COVID-19 pandemic [24]. The study employed a multi-modal recruitment strategy from September 2020 to December 2021 to ensure a racially and economically diverse sample from the counties of Alameda, Contra Costa, and San Francisco in California. These strategies included snowball sample recruitment through social media, engagement of existing users of the Streetwyze platform, and word-of mouth. We additionally partnered with a local, trusted community health center to facilitate recruitment through physical flyers and postcards and virtual presentations to existing community meetings. Qualitative data were collected using the Streetwyze mobile mapping and SMS platform, which was designed to allow community members to share real-time data on their experiences and perceptions of their neighborhoods [25]. On the platform, participants had the opportunity to view other user’s mapped stories and share their own experiences through ratings and open-ended reports on neighborhood amenities and destinations. Though audio, visual, and text options were available, all reports received for this study were text. Study participants were given prompts to focus responses, which were developed by community partners based on their “on-the-ground” assessment of relevant topics and to maximize benefit to community members (e.g., “What social or community services are you leaning on? Where are they located and what do you like/dislike about them?” and “Where are the places/spaces you are being treated with the most dignity and respect? What resources are helping your community survive and thrive during this challenging time?”). A full list of prompts is included as supplemental materials. Informed consent was obtained from all participants included in this study, and ethical approval was granted by the Institutional Review Board at the University of California, San Francisco.

### Data Analysis

**Fig. 1:**
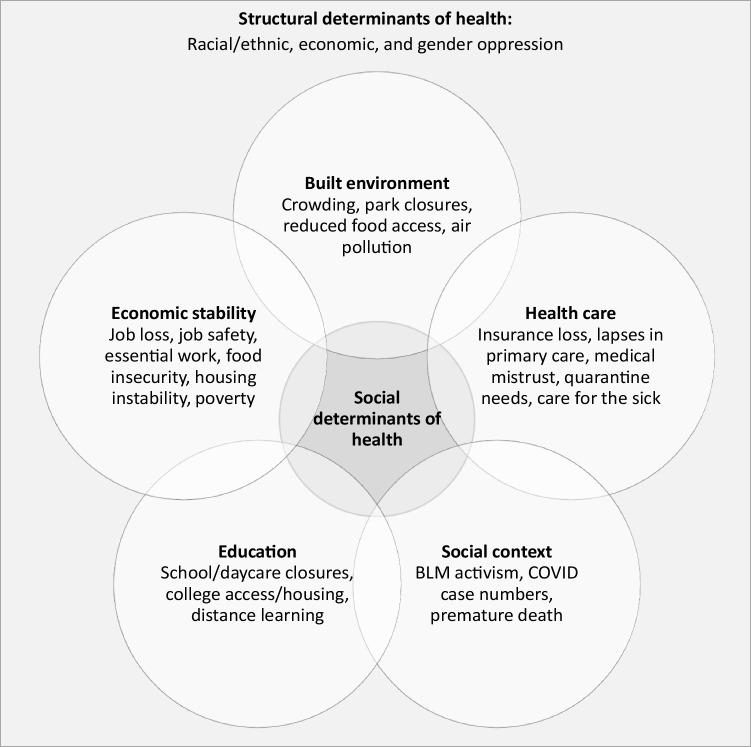
Structural and Social Determinants of Health, adapted from Healthy People 2020 [26]

Our qualitative methodology followed a constructivist research paradigm, aiming to explore and comprehend the lived experiences and perceptions of participants within the context of the COVID-19 pandemic. An initial codebook was developed based on hypothesized structural and social determinants of health that would be impacted or exacerbated by the COVID-19 pandemic (Fig. 1). Hypothesized domains in the initial codebook included factors relevant to COVID-19 infection control, resource needs, access needs, infrastructure needs, and well-being. Additionally, in this first stage of codebook development, two researchers read through all reports and made observations and notes to build upon the hypothesized themes and identify emergent factors not previously hypothesized or bounded by preconceived categories [27]. To achieve saturation, we iteratively employed the evolving codebook. Initially devised on a test sample, it was systematically applied to successive samples, refining emerging themes while incorporating new insights. This process underscored comprehensive coverage within the explored dataset, ensuring a robust exploration of qualitative data. After this initial codebook was developed, two researchers applied it to a random selection of reports to assess internal consistency in application of codes and to continue reiteration during the coding process. This codebook served as a living document that guided the following phases of qualitative analysis, while remaining flexible to revisions as it was applied to qualitative reports, such that if new codes emerged, all prior reports were analyzed to identify text for this code. All reports were coded by one academic researcher (A.G., M.D.) and one community researcher (T.C., B.M., and two additional researchers at Roots Community Health Center), all of whom had prior experience with conducting qualitative research. This data analysis team met several times over the course of the coding process, and all disagreements in coding were resolved through consensus. We employed a largely inductive approach to analysis to allow findings to emerge from the data without restraint [28].

## Results

A total of 236 qualitative neighborhood reports were collected from 19 respondents (median of 11 reports per participants). Most of the participants were Alameda County residents (*n*=15), under 45 years old (*n*=14), and female (*n*=12) (Table 1). Additionally, 16 participants were Asian American, Black, or Hispanic and 15 reported 6 months or less of savings in case of emergency. The most frequent business categories reported upon were non-food retail (*n*=46 reports), grocery (*n*=42 reports), restaurant (*n*=38 reports), and health care (*n*=28 reports) (Table 2).

**Table 1:** Characteristics of participants (*n*=19)

Characteristic	*N*	%
County		
Alameda	15	83
SF or Contra Costa	3	17
Age range		
18–44	14	78
45 and older	4	22
Race		
Asian American	5	26
Black	7	37
Hispanic	4	21
White/Other	3	16
Gender		
Male	7	37
Female	12	63
Student	6	32
Amount of money saved		
Less than a month	4	22
1–2 months	7	39
3–6 months	4	22
More than 1 year	3	17

**Table 2:** Characteristics of qualitative reports (*n*=236)

Characteristic	*N*	%
Business type or category		
Childcare	5	2.1
Church	4	1.7
Community food resources	9	3.8
Education	8	3.4
Election	7	3.0
Grocery	42	17.8
Health care	28	11.9
Innovation	3	1.3
Neighborhood	5	2.1
Other	10	4.2
Outdoor space	13	5.5
Recreational	4	1.7
Restaurant	38	16.1
Retail	46	19.5
Social services	6	2.5
Transportation	8	3.4
Review type		
Positive review	192	81.4
Negative review	14	5.9
Review for something to be fixed	29	12.3
Quarter when data was collected		
Sep–Dec 2020	132	55.9
Jan–Mar 2021	31	13.1
Apr–Jun 2021	12	5.1
Jul–Sep 2021	28	11.9
Oct–Dec 2021	28	11.9

Three themes emerged from this work. First, participants described innovative strategies that individuals employed or utilized to foster community cohesion and build informal networks. Second, participants spoke to the importance of tailored programming and services. Finally, participants articulated the importance of positive interactions with social service providers and quality of goods and services for dignified social services (e.g., health care, food).

### Theme #1: Innovation to Foster Community Cohesion and Establish Informal Networks

Participants described innovative strategies for building community and informal networks through sharing goods, resources, and knowledge. Some of these strategies included leveraging informal networks to access food resources, with one participant mentioning a “small community refrigerator [where] people can drop off extra food or pick up food here. They usually have bread, fruits and vegetables, and canned goods” (Asian female, under 45). Another mentioned, “I get free food from [this restaurant] because my friend works here” (Black male, over 45). Both examples speak to the power of mutual aid and networking to ensure that individual’s food needs were taken care of.

Another participant described their experience leveraging formal food services to obtain other types of goods within a black market operated by people experiencing homelessness in San Francisco:

The black market operates here [at this location]. I trade shelter in place bags of potato chips, 24 bags, for breakfast cereal. I buy instant coffee with food stamps and trade the coffee for clothes. However, note that this isn't the place for timely trades. If you have money, you can ask for what you want, then wait a day or so to get whichever it is. Food isn't like this. Bakery goods have a short shelf life, so does a dinner from the night before. My shelter in place hotel lost my clothes/laundry. SO, I'm looking for a trade partner, to buy some garments...in exchange for $100 worth of food*.*

- *White male, over 45*

Not only does this participant’s experience speak to ingenuity in maximizing utilization of available goods, but it also speaks to unspoken rules of an informal economy related to value and currency of items. The same participant also described the importance of sharing learned experiences and knowledge through informal networks, stating, “I met a woman on [location] who was really disconnected from the resource networks in SF… I brought her to [resource center] and helped connect her to a variety of resources at this center which consolidates them in one place.”

### Theme #2: The Value and Importance of Racial, Ethnic, and Culturally Tailored Services

Participants also described the importance of racially, ethnically, and culturally tailored programming and services during the pandemic. Some simply referred to specific communities being serviced, including comments such as “[Program name] has an amazing program for preschoolers. It really helps the Latino community” (Latino female, under 45) and “This is an amazing non-profit organization providing primary care services to the whole community regardless of legal status or insurance” (Latino male, over 45). Both participants indicate value in ensuring inclusivity in services. Finally, though we did not explicitly sample sexual or gender minority individuals, several participants alluded to services for this community. One participant described a social service organization as an “excellent community organization providing legal immigration services for LGBTQIA+ immigrants” (Latino male, over 45). Another expanded on specific services for individuals marginalized by both heterosexism and racism. They state:

Could not recommend this place more to people part of the LGBTQIA2S+ community and supporters. I rely on this place a lot to get dinner for my family… There are also events they have here like poetry slam night and dance classes or painting to uplift BIPOC voices and create a sense of community. They have the flags right in their window and also drive around and drop off the free dinners to those who call and need the assistance. The people who work there are kind, don't skip out on going here if you are in need of basic resources, it can take a lot of worry off your back*.*

- *Black female, under 45*

Both participants point to the importance of not only acknowledging the needs of communities experiencing intersectional oppression but ensuring that services are intentional about ensuring that multiple marginalized communities feel welcome to participate. As this participant described, the impact of doing so helped to ameliorate the stresses of life.

### Theme #3: Dignity in Service

Another major theme was the importance of dignity in social services across multiple domains. Two distinct characteristics emerged in participant’s descriptions of dignity: first, participants described respect and dignity in the *context of their interactions with service providers* (including healthcare providers, social service staff, and volunteers). For instance, as one participant described, “[This center] is the place to go if you’re looking to pick up bags of food for you or your family… They make sure to treat anybody in bed with dignity and respect” (Black female, under 45). Another participant expanded upon specific elements of their interaction that made them feel respected. Reporting on their experience receiving health care, this participant stated that, “[This doctor] provides the best care you could ask for. The staff is so nice and friendly. They make you very comfortable and treat like a family” (White female, under 45). While the theme of dignity and respect in service has been more commonly observed in healthcare and social service settings, participants in our sample referenced this in retail spaces as well. As one participant stated, “This grocery store takes EBT. Their products are a bit pricier, but they have very good safety standards. The employees at this location are very friendly and treat the customers very well, with both dignity and respect” (Latino female, under 45). This participant’s statement speaks to the ways in which impacts of stigma due to classism can be minimized in the retail setting. In general, participants’ reports of this theme across settings (e.g., crisis centers, healthcare centers) and types of services suggest that this was universally important.

Though less commonly described in our sample, some participants mentioned *the significance of quality goods as an element of dignity in service*. Sentiments about the quality of goods included that “the food that was given wasn’t expiring but stayed fresh for a long time” and “great place if you are in the need for cheap clothing. Not always the best quality, but affordable if you need it.” While not explicitly stated by these two participants, the impact of this tradeoff was described by another participant, in their experience with receiving free food services:

A lot of the food here is soupy and wears on your soul. Instead, I wish they had whole pieces of food, like chicken, that's fresh. When cooks put love in the food, it touches your heart. And when you're at the bottom of the bucket, these things really matter in the quality of your life. It peaks your spirit up, in times of crisis. [These two organizations are] where you can get food that looks and tastes like food*.*

- *White male, over 45*

Altogether, these participants’ statements allude to a tension and tradeoff between affordability (either with free or purchased goods) and the quality of the goods, which can have lasting impacts on an individual’s overall wellbeing.

## Discussion

Socioeconomic and racial inequities in socioeconomic and structural factors, such as health care, social context, and education are fundamental causes of health disparities [29, 30]. While these inequities existed long before the pandemic, they have been exacerbated across all levels of the eco-social model. Our study, which sought to highlight resources and strategies residents used to promote health and well-being during the pandemic, uncovered several unique themes. First, we found that participants leveraged goods, services, and learned experiences to create informal networks and share resources with one another. Second, participants described the use and value of programming that was tailored to the unique needs of groups experiencing multiple marginalization. Third, participants noted several important elements that could foster a sense of dignity when receiving social services, including the value of positive interactions with service providers and the quality of goods.

Our findings on the value and importance of informal networks and resource sharing suggest that improvement in existing services and the promotion and investment in mutual aid programs can help to fill the gaps in resource needs experienced by racially and economically marginalized communities. For instance, participants described the suitability of mutual aid for delivering food resources. However, mutual aid is not a new concept and has been applied to a wide variety of contexts, from behavioral health to economic and social support [31]. For racially and economically marginalized communities, mutual aid has been found to be an effective tool for social movement and community building [32]. A key exemplar of the success of mutual aid programs is the Black Panther’s free breakfast program, which fed thousands of children before school and was co-opted into a government program [33]. Not only have mutual aid programs been found to be beneficial to fill gaps in resource needs, but they are also practical to promote belonging and social support. Altogether, investment in community-centered sufficiency and mutual aid programs can be a promising approach to minimizing racial and economic disparities.

None of our findings can be disentangled from the backdrop of social, political, economic, and racial tensions that were contemporaneous with the pandemic. For instance, the substantial number of excerpts that expressed the importance of tailored programming and need for racially, ethnically, and culturally specifical services occurred alongside massive demonstrations for Black Lives Matter [34], discourse surrounding the upsurge in nationwide anti-Asian violence following associations of Asian people with the origins of COVID-19 and the Atlanta massacre [35, 36], and the insurrection at the United States Capitol [37]. Similarly, descriptions of dignity across categories—from health care to retail—are inextricably linked to long-standing stigma associated with poverty [38, 39], and the important function of community-based organizations in promoting high-quality care while minimizing harm. Any future interventions that seek to promote equity and justice for racially and economically marginalized communities must include considerations of the social and political context to be meaningful, sustainable, and transformative. For instance, participants in our study mentioned several tangible solutions for increasing inclusivity and belonging, including the obvious display of signs, racial and ethnic concordance between service providers and community members, tailoring of services for groups experiencing intersectional oppression, and offering various mechanisms for service delivery (e.g., delivery of basic needs upon request).

Finally, in addition to the emergent themes described, we found mentions of other sentiments reflective of society at-large during the pandemic. These included mentions of the built environment (e.g., descriptions of noise, trash, or general disorder in a space) which have been found to be important for health and well-being. Similarly, mentions of outdoor spaces, trails, greenspaces, and parks were common across qualitative reports, illustrative of the ways in which joy in outdoor spaces was reimagined during the pandemic. Finally, another common theme across qualitative reports was the convenience and availability of goods and services, which itself can be reflective of the evolution of access throughout the course of the pandemic. In combination, all these common sentiments were reflective of the larger societal experience of COVID-19 in the USA. While our data precludes the ability to draw conclusions about potential differences in these factors along the gradient of privilege and oppression, there is already extensive evidence of disparities in positive built environment characteristics, outdoor spaces, and access to goods and services for racially and economically marginalized communities. Therefore, the prevalence of these factors in our study provides evidence for the continued and potentially exacerbated effects of these disparities because of COVID-19.

This article contributes to the growing landscape of research which qualitatively describes the resource needs and coping strategies that racially and economically marginalized communities employed during the COVID-19 pandemic [21, 40–42], specifically in the context of the San Francisco Bay Area. The study design and use of a mobile data collection platform allowed individuals to participate as they moved within their daily lives, thus providing more flexibility for engaging in qualitative research. Because the mobile platform was shared by participants, they could benefit from shared community knowledge. Furthermore, our inductive thematic analysis ensured that results interpretation was grounded in participants’ lived experiences and minimized researcher bias. That said, we note some limitations. Limitations in the generalizability of the study findings arise from the restricted sample size and its composition. Primarily consisting of younger individuals, with only three participants aged 45 or above, the study’s demographic representation may not fully encompass broader age groups. Additionally, the use of short-form data restricted the researchers’ ability to engage in further in-depth probing, potentially limiting the thorough exploration of enduring themes. Furthermore, given the study’s short-term nature, capturing dynamic changes in resources and needs over an extended period was not feasible. While an iterative coding approach was utilized for thorough analysis of participant submitted texts, it should be noted as a potential limitation that participant feedback or corrections were not sought. However, the active involvement of community partners in the coding process likely contributed to ensuring the relevance and accuracy of interpretation.

Pre-pandemic levels of inequity among racially and economically marginalized communities have been a top priority for public health and policy leaders alike. These inequities have only worsened over the course of the pandemic [43–45]. In this qualitative paper, we described several themes which speak to strategies that have been employed to deal with the persistent inequalities, which were exacerbated by COVID-19. While these themes and strategies are certainly not new, they seem to be particularly relevant during the pandemic, and will continue to be important as other macro-level stressors (e.g., climate stress, sociopolitical tension) become more influential in the coming decades. In conclusion, this study underscores the urgency of bolstering mutual aid networks, prioritizing culturally sensitive services, and championing dignified care within social services for marginalized communities. As we navigate evolving challenges, the imperative lies in conducting longitudinal research to assess the dynamic interplay of stressors and translating these insights into impactful policies and programs that foster equity and empower resilient communities.

## Supplementary Information

ESM 1 (PDF 138 KB)

## Data Availability

Data for this study are not publicly available as they contain information that could compromise the privacy of research participants.
