# Supplementary material for: Dignified Resources and Coping Strategies During the COVID-19 Pandemic: a Qualitative Study of Racially and Economically Marginalized Communities
Source: J Racial Ethn Health Disparities. 2023 Oct 16;11(6):3748–56. doi: 10.1007/s40615-023-01824-x (PMC11564376; doi:10.1007/s40615-023-01824-x)
Supplement: Supplementary file 1 — (PDF 138 KB) [file 40615_2023_1824_MOESM1_ESM.pdf]

SUPPLEMENTAL MATERIALS
Journal of Racial and Ethnic Health Disparities

Dignified Resources and Coping Strategies During the COVID-19 Pandemic: A Qualitative Study of Racially and Economically Marginalized Communities

Alice Guan, Tessa Cruz, Jamaica Sowell, Brenda Mathias, Analena Hope Hassberg, Salma Shariff-Marco, Antwi Akom, Mindy C. DeRouen

Prompts for qualitative data collection:
The following questions were offered as prompts to participants who were completing neighborhood reports through the Streetwyze application:

- How and where are you accessing health care at this time? What is experience like accessing the health support you need?c
- Where is COVID testing available? What hours and for whom? Where are they located and what do you like/dislike about them
- Where are you finding groceries? Toilet paper? Soap? How long are the lines?
- Where are you finding resources?
- Where is the safest and most affordable for you to go, to get the things you need?
- What resources are unavailable/hard to find?
- How are you being treated?
- Are there social or community services you are leaning on that you would like to lift up? If so, what are they? Where are they located and what do you like/dislike about them?
- Are there community-based organizations or neighbors who are making a difference in your life that you'd like to highlight?
- What are you doing to protect their health and support your community during COVID?
- Are there any helpful "tips" or "tricks" you'd recommend to others for navigating life outside your home (if/when you have to leave home for essential services)?
- Are there services or places or you think could be improved in times of crisis like this? If so, how?
- What about women and children? What places are taking WIC/EBT?
- What places do feel safe/unsafe going? Why?
- Where are the places/spaces you are being treated with the most dignity and respect? What resources are helping your community survive and thrive during this challenging time?
- Which busses/trains are running on time that can be counted on during this time?
- What social or community services are you leaning on? Where are they located and what do you like/dislike about them?
